# Attitudes to mental illness among mental health professionals in Singapore and comparisons with the general population

**DOI:** 10.1371/journal.pone.0187593

**Published:** 2017-11-16

**Authors:** Qi Yuan, Louisa Picco, Sherilyn Chang, Edimansyah Abdin, Boon Yiang Chua, Samantha Ong, Kah Lai Yow, Siow Ann Chong, Mythily Subramaniam

**Affiliations:** 1 Research Division, Institute of Mental Health, Singapore, Singapore; 2 Nursing Administration, Institute of Mental Health, Singapore, Singapore; 3 Clinical and Allied Health Professionals Services, Institute of Mental Health, Singapore, Singapore; Universidade Federal do Rio de Janeiro, BRAZIL

## Abstract

**Background:**

Similar to the general public, mental health professionals sometimes also have negative attitudes towards individuals with mental illness; which could ultimately affect the quality of care received by the patients. This study aims to explore attitudes to mental illness among mental health professionals in Singapore; make comparisons with the general population; and investigate the significant correlates.

**Methods:**

A cross-sectional design was used. Eligible participants were recruited from the Institute of Mental Health, Singapore. Attitudes to mental illness among the mental health professionals were measured using an adapted 26-item Attitudes to Mental Illness questionnaire (AMI). An earlier study amongst the general population in Singapore had used the same tool; however, factor analysis suggested a 20-item, 4-factor structure (AMI-SG) was the best fit. This 4-factor structure was applied among the current sample of mental health professionals to allow comparisons between the professionals and the general population.

Data were collected through an online survey tool ‘Questionpro’ from February to April 2016, and 379 participants were included in the current analysis. Attitudes to mental illness among these professionals were compared to those of the general population, which were captured as part of a national study conducted from March 2014 to April 2015.

**Results:**

The 20-item, 4-factor structure AMI-SG derived from the general population was applicable among the mental health professionals in Singapore. Compared to the general population, mental health professionals had significantly more positive attitudes to mental illness; however their scores on ‘social distancing’ did not differ from the general population. Indian ethnicity was negatively associated with ‘social distancing’ and ‘social restrictiveness’ among the professionals; while higher education was negatively related to ‘prejudice and misconception’. Compared to nurses, doctors showed significantly more positive attitudes on ‘social restrictiveness’ and ‘prejudice and misconception’. Having family or close friends diagnosed with mental illness was negatively associated with ‘social distancing’ among the professionals.

**Conclusion:**

The AMI-SG is an effective tool to measure attitudes to mental illness among mental health professionals in Singapore. Although the professionals had significantly more positive attitudes to mental illness than the general public in Singapore, their attitudes on ‘social distancing’ resembled closely that of the general public. Professionals tended to have more negative attitudes if they were nurses, less educated, and of Chinese ethnicity. More studies are needed to explore the underlying reasons for the differences and to generalize these findings among mental health professionals elsewhere.

## Introduction

Attitudes to mental illness can encompass positive attitudes such as acceptance [1], more neutral attitudes like tolerance [2], to negative ones such as stigma [3] and even fear [4]. Previous studies suggested that negative attitudes towards people with mental illness were highly prevalent among the general public [5–8]. Similarly, mental health professionals may also have negative attitudes towards people with mental illness. A previous study in Brazil found that among a national sample of 1,414 psychiatrists, 42.9% of them were identified as having stereotype, prejudice and high social distance towards individuals with schizophrenia [9]. Nordt et al. [10] also found mental health professionals had similar level of negative attitudes about people with mental illness as the general public. Hansson et al. [11] examined attitudes of patients and mental health professionals towards people with mental illness and found that there was a high prevalence of negative attitudes towards people with mental illness among the mental health professionals, and the professionals’ beliefs about people with mental illness were similar to those of the patients.

Negative attitudes among mental health professionals might affect patients in multiple ways. For example, Ellsworth [12] found that nurses’ higher endorsement on restrictive attitudes were consistently related to more controlling and restricting behaviours among the nurses towards the patients. Other than the behaviours, negative attitudes have also been shown to affect the therapeutic alliance between professionals and patients [13]. A study done by Verhaeghe and Bracke [14] reported that mental health service users with more stigma experience during the service tended to show less trust and less satisfaction towards the service. Previous studies also suggested that psychiatric nurses could have negative attitudes towards specific mental disorders (e.g. borderline personality disorder [15]) and towards individuals with mental illness in general [16, 17]. However, nurses’ attitudes are one of the most important factors in facilitating progress movement within the one-to-one therapeutic relationship [18, 19]. In all these circumstances, attitudes among the professionals could ultimately lower the quality of care received by the patients.

Despite some research highlighting the harmful impact these negative attitudes can have towards people with mental illness, others also suggested that mental health professionals, due to their knowledge and daily contact with individuals with mental illness, would have more positive attitudes towards mental illness. Mårtensson et al. [20] found that compared to the general Swedish population, mental health nurses in Sweden had more positive attitudes to mental illness. Similar findings were also reported by Lauber et al. [21] who suggest that psychiatrists have significantly more positive attitudes to mental illness compared to the general population. Compared with non-mental health professionals, mental health professionals also possess more positive attitudes towards individuals with mental illness [22–24]. However, these studies all have their own limitations, such as small sample size [21–24] and poor analysis strategy (i.e. comparisons without controlling for potential confounding) [20, 23, 24]. Moreover, due to the inconsistent results of such comparisons, more studies are still needed in this area.

Unlike various studies comparing attitudes of mental health professionals with other healthcare professionals [22–24] or general public [10, 11, 20, 21], fewer studies have been done to explore correlates of attitudes to mental illness among mental health professionals. For example, Mårtensson et al. [20] found that nursing staff had more positive attitudes towards mental illness if their knowledge about mental illness was less stigmatized (i.e. higher endorsement on the six stigma related statements of the Mental Health Knowledge Schedule [25]), and if they had a friend with mental illness currently or in the past. Another study by Hansson and colleagues [11] suggested that mental health professionals’ attitudes towards mental illness might be affected by their work setting characteristics, with staff working within inpatient services having more negative attitudes than those working in out-patient services. Cross-cultural studies on attitudes to mental illness among mental health professionals also suggest that cultural differences might contribute to the differences in attitudes. A previous study which compared Swiss mental health professionals with counterparts in Brazil showed that professionals in Switzerland had significantly higher levels of social distance, and age was a statistically significant predictor (i.e. younger age associated with less social distance) [26]. Another study among mental health nurses across 5 European countries (i.e. Finland, Lithuania, Ireland, Italy and Portugal) also suggested the attitudes differed by country of origin [27]. To the best of our knowledge, there is only one study which was conducted among Singapore mental health professionals to explore their attitudes to mental illness [28]. However, that study was conducted more than 10 years ago and the study sample only included nurses. Moreover, the bivariate analysis strategy used in that study could not exclude the potential confounding effects of other variables.

In order to address these gaps, the current study aims to 1) explore attitudes to mental illness among mental health professionals in Singapore; 2) compare the attitudes of mental health professionals with the local general population; 3) explore the correlates of attitudes to mental illness among mental health professionals.

## Methods

### Participants and procedure

Data relating to attitudes of mental health professionals were extracted from a study which aimed to explore associative stigma and positive mental health among staff working at the Institute of Mental Health (IMH), the only tertiary psychiatric service provider in Singapore [29]. This was a cross-sectional, online survey which used convenience sampling. Inclusion criteria comprised being: 1) Singapore citizens, Permanent Residents or Non-residents; 2) doctors, nurses, or allied health staff (i.e. psychologists, pharmacists, occupational therapists, physiotherapists, case managers or medical social workers) working at IMH during recruitment; 3) aged 21 years and above and; 4) able to complete the online survey in English. Given that we also aimed to compare the attitudes to mental illness of the mental health professionals with that of the general population in Singapore, data from non-residents or work permit holders were excluded from the final analysis, to ensure comparability.

Data were collected from February to April 2016. Email invitations were sent to all nurses, doctors, and allied health staff through relevant hospital group email lists informing them of the study. By clicking the link enclosed in the email, potential participants were directed to an online portal including the screening questions. They were used to ensure the respondents met the inclusion criteria. For those who were screened out, an automatic email was sent notifying them that they were not eligible for this study. The remaining participants were then directed to the online consent form. Clicking the ‘agree’ button indicated an individual’s willingness to participate in the study. The questionnaire was administered through an online survey tool ‘Questionpro’, and took around 10–15 minutes to complete. Upon completion, a SGD$20 Starbucks voucher was given to the participants to compensate for their time and inconvenience. In total, 470 participants were recruited during the 2-month recruitment period, among which 8 cases were removed due to unreliable data or the respondents not meeting the inclusion criteria and another 83 cases were excluded from analysis due to them being non-residents. In all, 379 mental health professionals were included in the current study. Ethical approval was granted by the National Healthcare Group Domain Specific Review Board, Singapore.

### Measurements

Attitudes to mental illness were measured by an adapted 26-item version Attitudes to Mental Illness questionnaire (AMI). The original AMI questionnaire has 27 items in total, and it was developed by the UK Department of Health [30, 31], based on the 40-item Community Attitudes toward the Mentally Ill Scale [32]. An earlier study conducted by the same study team had also used the AMI questionnaire among a national representative sample of the general population in Singapore [33]. Factor analysis of this 26-item AMI questionnaire revealed a 20-item, 4-factor structure which was the best fit for the general population in Singapore, and comprised: ‘social distancing’ (3 items), ‘tolerance/support for community care’ (9 items), ‘social restrictiveness’ (3 items) and ‘prejudice and misconception’ (5 items) [33]. Given the current study aimed to compare attitudes to mental illness between mental health professionals and the general population in Singapore, whilst the 26-item version of the AMI questionnaire was administered, analysis was based on the 20-item, 4-factor version. Henceforth, to differentiate these two versions, the 20-item version is referred to as the Attitudes to Mental Illness questionnaire—Singapore version (AMI-SG). The 20 items of the AMI-SG can be found in the S1 Appendix.

The participants were required to rate items on a 5-point Likert scale ranging from ‘1 = strongly agree’ to ‘5 = strongly disagree’. To enable easier interpretations, the AMI-SG items were reverse scored (changed to ‘1 = strongly disagree’ to ‘5 = strongly agree’) as per an earlier national study conducted in Singapore [33]. The total score of each factor was then summed and used in the multivariate regression analyses. For tolerance/support for community care, the reverse-scoring item ‘Increased spending on mental health services is a waste of money’ was reverse scored again, and then added up with the scores of the remaining items within this factor. As a result, more positive attitudes towards people with mental illness were characterized as—lower ‘social distancing’, ‘social restrictiveness’ and ‘prejudice and misconception’ and higher ‘tolerance/support for community care’ scores.

Socio-demographic information including age, gender, ethnicity, marital status, education level, residency status, and information on employment (i.e. position and years worked in IMH), and whether any close family and friends had been diagnosed with mental illness was also collected.

### Attitudes to mental illness among general population

Data on attitudes to mental illness among the Singapore general population (i.e. socio-demographic characteristics including age, gender, ethnicity, marital status, education level, and AMI-SG factor scores) were extracted from a previous national mental health literacy study conducted from March 2014 to April 2015 [33]. Since the main purpose of the current study was to explore attitudes to mental illness among mental health professionals, and the fact that detailed information of this national study could be found elsewhere [33]; in the current study, this extracted data were purely used in the comparison analysis.

### Statistical analysis

Descriptive analyses were performed for socio-demographic and other dependent variables. Continuous variables were listed as mean and standard deviation (SD); for categorical variables, they were presented as frequency and percentage. Before further analysis, a confirmatory factor analysis (CFA) was conducted, to test the applicability of the 20-item, 4-factor AMI-SG derived from the general population in Singapore, among the current sample of mental health professionals, whilst also ensuring the construct validity [34] of AMI-SG among the mental health professionals before further comparisons. CFA was performed through the ‘lavaan’ package under R software [35], and adjusted for categorical variables with the estimator of ‘Weighted Least Square Means and Variance Adjusted (WLSMV)’ [36]. In the current study, an acceptable model was defined as 1) the comparative fit index (CFI) > 0.90; 2), the Tucker-Lewis index (TLI) > 0.90, and 3) the root mean square error of approximation (RMSEA) < 0.08 [37]; while for a good model, these indices should be ‘CFI > 0.95, TLI > 0.95 and RMSEA < 0.06’ [38]. The internal consistency indicators (Cronbach’s alpha) were also calculated for each factor.

To compare the AMI-SG factor scores between the general population and mental health professionals in Singapore, the two datasets were combined with the included variables being recoded to ensure consistency across the two datasets. Given the study among the general population adopted a disproportionate stratified sampling design with 12 strata defined according to ethnicity (Chinese, Malay, Indian, Others) and age group (18–34, 35–49, 50–65 years) [33]; during the combination, a separate strata number (i.e. 13) and a weight equals to ‘1’ (to indicate no sampling weight) were assigned to the mental health professionals sample. Multivariate regression was conducted with each of the AMI-SG factors being the dependent variable and mental health professionals or general population as the independent variable, after controlling for socio-demographic characteristics including age, gender, ethnicity, marital status and education level. This analysis was conducted via the ‘PROC SURVEYREG’ syntax in SAS9.3 [39]. The mean AMI-SG factor scores of the general population were also extracted from Yuan et al. [33].

Lastly, multivariate linear regression was performed to examine the significant correlates (e.g. socio-demographic characteristics such as age, gender and ethnicity, position, years worked at IMH and whether any close family and friends had been diagnosed with mental illness) of each of the AMI-SG factor scores (dependent variables) among the mental health professionals working at IMH. The descriptive analysis and this multivariate linear regression analysis were also conducted using SAS 9.3 [39]. For all regression analyses, a two-sided p-value below 0.05 was considered as statistically significant.

## Results

The sample characteristics are presented in Table 1. The sample (n = 379) comprised 51 doctors, 137 nurses, and 191 allied health staff. The average age of the study sample was 37.4 (SD = 11.0) years, with the majority being female (65.7%), Chinese (70.5%), having a bachelor’s degree or above (84.2%), and having worked at IMH for at least one year (91.6%). Only 30.9% participants reported that they had family or close friends who have been diagnosed with mental illness.

**Table 1:** Socio-demographic characteristics of the sample (n = 379).

Characteristics	Groups	Mean/Frequency	SD/%
**Age (years)**		37.4	11.0
**Gender**	Female	249	65.7
	Male	130	34.3
**Ethnicity**	Chinese	267	70.5
	Indian	53	14.0
	Malay	36	9.5
	Others (including Filipino and Myanmar)	23	6.1
**Marital status**	Ever married	216	57.0
	Never married	163	43.0
**Education level**	Secondary/ITE/'O' level	18	4.8
	A' level/diploma	42	11.1
	Bachelor	173	45.7
	Master or above	146	38.5
**Nationality**	Singapore Citizen	320	84.4
	Permanent Resident	59	15.6
**Position**	Doctors	51	13.5
	Allied health staff	191	50.4
	Nurses	137	36.2
**Years in IMH**	Less than 1 year	32	8.4
	1–5 years	148	39.1
	6–10 years	88	23.2
	More than 10 years	111	29.3
**Family or close friends diagnosed with mental illness**	Yes	117	30.9
	No	262	69.1

The CFA results confirmed that the 4-factor structure of AMI-SG derived from the general population in Singapore [33] was acceptable among mental health professionals working at IMH. The fit indices were χ^2^_(df)_ = 485.086 _(164)_ (p = 0.000), CFI = 0.930, TLI = 0.919, RMSEA = 0.072. The absolute value of the factor loading for each item varied from 0.451 to 0.956. The internal reliability statistics for the four factors, namely ‘social distancing’, ‘tolerance/support for community care’, ‘social restrictiveness’ and ‘prejudice and misconception’, were 0.791, 0.735, 0.663, and 0.671, respectively; where a cut-off of 0.6 was deemed as acceptable [40]. The mean AMI-SG factors scores among the mental health professionals were 7.52 for ‘social distancing’, 40.48 for ‘tolerance/support for community care’, 5.16 for ‘social restrictiveness’, and 11.20 for ‘prejudice and misconception’ (Table 2).

**Table 2:** AMI-SG factor scores among mental health professionals and general population in Singapore.

	Mental Health Professionals		General Population	Score Range
	Mean	*SD*	Mean	
**Social Distancing (3 items)**	7.52	2.97	8.07	3–15
**Tolerance/Support for community care (9 items)**	40.48	4.46	14.81	9–45
**Social Restrictiveness (3 items)**	5.16	2.12	7.21	3–15
**Prejudice & Misconception (5 items)**	11.20	3.94	15.36	5–25

Table 3 shows the comparisons of AMI-SG factor scores between mental health professionals and the general population in Singapore. The results suggest that, after controlling for socio-demographic characteristics including age, gender, ethnicity, marital status and education level, mental health professionals had significantly more ‘tolerance/support for community care’, less ‘social restrictiveness’, and less ‘prejudice and misconception’, compared to the general population. However, ‘social distancing’ scores did not differ between the two groups.

**Table 3:** Comparison of AMI-SG factor scores between mental health professionals and general population in Singapore.

	Social Distancing			Tolerance/Support for community care			Social Restrictiveness			Prejudice & Misconception		
	β	95% CI		β	95% CI		β	95% CI		β	95% CI	
**Mental health professionals**	-0.258	-0.629	0.113	26.345*	25.790	26.900	-1.191*	-1.488	-0.893	-2.220*	-2.686	-1.755
**General population**	Ref			Ref			Ref			Ref		

Ref—reference group;

* p<.0001;

Controlled for socio-demographic characteristics including age, gender, ethnicity, marital status and education level;

Multivariate linear regression results for all 4 AMI-SG factors among the mental health professionals working at IMH are presented in Table 4. The results revealed that Indians and those from ‘other’ ethnic groups (including Filipino and Myanmar) as well as those who had family or close friends diagnosed with mental illness, showed significantly less ‘social distancing’ towards individuals with mental illness; while mental health professionals who were ever married showed significantly more ‘social distancing’ towards people with mental illness. ‘Tolerance/support for community care’ was only correlated with education level, with individuals with Secondary/ITE/'O' level education having significantly lower level of ‘tolerance/support for community care’ for mental illness. ‘Social restrictiveness’ was negatively associated with being Indian and being a doctor. While ‘prejudice and misconception’ toward people with mental illness was positively associated with lower education level and being a permanent resident, as well as negatively associated with being either a doctor or allied health staff.

**Table 4:** Correlates of AMI-SG factor scores among mental health professionals.

	Social Distancing				Tolerance/Support for community care				Social Restrictiveness				Prejudice and Misconception			
	β	95% CI		p	β	95% CI		p	β	95% CI		p	β	95% CI		p
**Age**	-0.014	-0.051	0.022	0.444	0.005	-0.051	0.061	0.851	-0.007	-0.033	0.019	0.593	-0.009	-0.049	0.032	0.674
**Gender**																
Female	-0.661	-1.332	0.009	0.053	0.453	-0.573	1.480	0.386	-0.223	-0.700	0.254	0.359	0.113	-0.624	0.851	0.762
Male	Ref				Ref				Ref				Ref			
**Ethnicity**																
Indian	-1.326	-2.280	-0.372	**0.007**	-0.653	-2.128	0.822	0.385	-0.811	-1.492	-0.129	**0.020**	-0.211	-1.260	0.837	0.692
Malay	-0.607	-1.811	0.596	0.322	0.898	-0.974	2.770	0.346	-0.519	-1.381	0.344	0.238	0.385	-0.955	1.724	0.573
Others	-1.705	-3.201	-0.210	**0.026**	-1.293	-3.579	0.993	0.267	-0.304	-1.368	0.761	0.575	0.707	-0.962	2.377	0.405
Chinese	Ref				Ref				Ref				Ref			
**Marital status**																
Ever married	**0.795**	**0.104**	**1.486**	**0.024**	-0.900	-1.964	0.164	0.097	0.128	-0.363	0.619	0.610	0.042	-0.717	0.801	0.913
Never married	Ref				Ref				Ref				Ref			
**Education level**																
Secondary/ITE/'O' level	0.028	-1.579	1.635	0.973	-4.582	-7.035	-2.130	**0.0003**	0.122	-1.019	1.263	0.834	5.070	3.303	6.837	<**.0001**
A' level/diploma	-0.566	-1.856	0.725	0.389	-1.693	-3.666	0.280	0.092	0.562	-0.368	1.491	0.236	4.120	2.695	5.545	<**.0001**
Bachelor	-0.045	-0.798	0.708	0.906	-0.907	-2.062	0.248	0.124	0.064	-0.470	0.597	0.815	1.537	0.711	2.364	**0.0003**
Masters or above	Ref				Ref				Ref				Ref			
**Residency**																
Permanent Resident	0.329	-0.660	1.317	0.513	0.939	-0.567	2.444	0.221	0.169	-0.540	0.877	0.639	1.450	0.360	2.540	**0.009**
Singapore Citizen	Ref				Ref											
**Position**																
Doctors	-0.470	-1.692	0.751	0.449	0.568	-1.303	2.439	0.551	-1.431	-2.299	-0.563	**0.001**	-2.338	-3.677	-0.999	**0.0007**
Allied health staff	-0.537	-1.435	0.361	0.240	0.085	-1.307	1.477	0.905	-0.427	-1.065	0.211	0.189	-1.880	-2.863	-0.897	**0.0002**
Nurses	Ref				Ref				Ref				Ref			
**Years in IMH**																
Less than 1 year	-0.656	-2.098	0.787	0.372	-0.630	-2.809	1.550	0.570	-0.128	-1.147	0.892	0.806	0.523	-1.054	2.100	0.515
1–5 years	-0.502	-1.488	0.484	0.317	-0.137	-1.645	1.372	0.859	-0.678	-1.379	0.022	0.058	-0.636	-1.735	0.462	0.256
6–10 years	0.071	-0.872	1.013	0.883	-0.788	-2.231	0.655	0.283	-0.363	-1.035	0.308	0.288	-0.596	-1.637	0.445	0.261
More than 10 years	Ref				Ref				Ref				Ref			
**Family or close friends diagnosed with mental illness**																
Yes	-0.947	-1.620	-0.275	**0.006**	0.813	-0.218	1.844	0.122	-0.303	-0.779	0.172	0.210	-0.321	-1.056	0.413	0.390
No	Ref				Ref				Ref				Ref			
**R Square**	0.096				0.081				0.102				0.385			

Ref—reference group;

## Discussion

The CFA analysis confirmed that the 4-factor structure of AMI-SG derived from the Singapore general population was applicable among mental health professionals in Singapore. It suggested that AMI-SG had good construct validity in measuring attitudes to mental illness among mental health professionals. Compared to the general population, the professionals had significantly more positive attitudes to mental illness—less ‘social restrictiveness’ and ‘prejudice and misconception’; and more ‘tolerance/support to community care’. This finding is consistent with those reported by the majority of the studies in the literature [20, 21, 41, 42]. However, for ‘social distancing’, no statistically significant difference was identified. Lauber et al. [21] reported that ‘although accepting mental health facilities in the community, psychiatrists also agree mental health facilities are downgrading a residential area’, and he named it as a minor form of the ‘not in my back yard’ phenomenon. Our study, on the other hand, provided more solid evidence for this phenomenon, suggesting that although mental health professionals tends to have more ‘tolerance/support for community care’, less ‘social restrictiveness’ and less ‘prejudice and misconception’, their desire for closeness or intimacy towards individuals with mental illness (‘social distancing’) resemble closely that of the general public.

Another interesting finding was how having ‘family or close friends diagnosed with mental illness’ affected the attitudes of the mental health professionals. Various studies have suggested that social contact is one of the most effective interventions to reduce mental health related stigma and discrimination among adults [43, 44]. This was also observed among the local general population, i.e. a previous national study suggested that social contact was negatively associated with personal stigma and social distance [6]. In our study, the results suggested that among professionals, having family or close friends diagnosed with mental illness (personal contact experience) predicted significantly less social distance towards those with mental illness. This finding, together with the difference in attitudes to mental illness between mental health professionals and the general population suggests that although both professional contact and personal contact belongs to social contact; the mechanism of how they work might be totally different. This assumption is supported by the intergroup contact hypothesis by Allport [45] where professional and personal contact represent the typical contact experience between groups with unequal group status (i.e. professionals and patients) and equal group status (i.e. friend and friend). However, due to the limitation of observational study design; more studies, especially qualitative and longitudinal studies, are needed to further test this assumption and to explore the exact underlying mechanism for such differences.

The demographic correlates of AMI-SG domains among mental health professionals included ethnicity, marital status, education level, and residency status. Indian ethnicity was negatively associated with social distancing and social restrictiveness. This is consistent with findings from a multi-national study suggesting that pharmacy students from India had lower level of social distancing compared to students from Australia, Belgium, Estonia and Latvia [46]. Permanent residents showed more prejudice and misconception towards mental illness compared to citizens. One possible explanation for this finding could be the different training received by permanent residents and local mental health professionals. Permanent residents would normally be trained in their own countries (e.g. India and China), which usually adopt a different system compared to that in Singapore (e.g. the undergraduate training in Singapore has an emphasis on communication skills [47], while this is not the case in India and China [48]). Such differences might lead to different values and perspectives among the professionals, and in turn result in different views towards individuals with mental illness. Since ethnicity and residency status both are relevant to culture, their correlations with AMI-SG indicate the potential role of culture as an underlying factor that can influence attitudes to mental illness. Mental health professionals who were ever married tended to show more ‘social distancing’ towards mental illness compared to those who were never married. However, since the potential effect of marriage on professionals’ attitudes was not tested among previous studies [10, 11, 20, 21], more empirical studies are still needed to confirm the relationship and explore the potential explanations. Regarding the finding on education level, for future attitude campaigns among mental health professionals, these should be designed and tailored towards those with lower education levels. Other factors like age and gender which were found to be significant predictors among the general population [33] did not have any effects on the AMI-SG domains among the mental health professionals.

The different occupations of the mental health professionals also contributed to differences in AMI-SG domain scores, with doctors showing significantly less ‘social restrictiveness’ and ‘prejudice and misconception’, while allied health staff also showed less ‘prejudice and misconception’ compared to nurses. This is quite surprising yet understandable. Two reasons might have led to such a phenomenon. First of all, the different roles of mental health professionals were determined largely by their training. Although all participants were working in a tertiary mental health hospital, a previous study suggested that more than half of the nurses working at IMH were not registered as psychiatric nurses [28]. Instead they received general nursing training before they started working at IMH, and then they received on-site training. While for doctors at IMH, they are normally trained specifically under psychiatry which focuses more on the mechanism of mental disorders and associated treatments. This enables doctors to have a better understanding of mental illness and the behaviours of the patients. Such difference allows the doctors to make more objective judgement over the patients and how they might perform in society. Thus it’s not surprising that doctors had less ‘social restrictiveness’ and less ‘prejudice and misconception’ towards individuals with mental illness. Another difference between doctors and nurses is that for doctors, their contact with patients is usually formalized and lasts for a certain period of time in the consultation room; this also applies to their contact with inpatients. For allied health staff, they are more supportive in nature compared to doctors and nurses; thus have relatively less direct contact with patients. Even for those whom need to interact with the patients, they mainly deal with short-stay patients whom are usually non-severe cases [28]. While for nurses, they work more in the inpatient settings (i.e. short- and long-stay patients) where they are heavily involved in the daily care of more severe patients; and these severe patients, compared to outpatients, are more likely to be less cooperative. According to Allport [45], intergroup contact would lead to favourable outcomes when the participants have equal status, common goals and intergroup cooperation. In this case, compared to doctors, nurses’ contact experiences are more likely to violate these conditions; which in turn result in less improvement in their attitudes. This is consistent with assumption from researchers, indicating that increased personal and professional contact is associated with more positive attitudes to people with mental illness [20]; however, contact with individuals with more severe, long-term and recurrent mental illness might lead to comparatively more negative attitudes [11]. This explanation is supported by the findings from a previous study on attitudes towards people with mental illness among nurses working at IMH, which found nurses working in the short-stay wards had more positive attitudes than those working in the long-stay wards [28]. In this case, it is important to determine the level of involvement with the inpatient care of people with mental illness which would produce the most optimum shift in nurses’ attitudes towards mental illness. For example, one could explore for nurses who are working in the long-stay wards, if providing more support to share their care responsibilities or having more rotations between short- and long-stay wards would improve their attitudes.

The current study has several strengths. Firstly, it is the first study comparing the attitudes to mental illness between the general public and the mental health professionals in Singapore. Secondly, the utilization of online tools during the data collection reduced efforts in data entry, and it also obviated the associated data entry errors. Moreover, the absence of interviewers during the data collection process also helped to avoid the social desirability bias. Lastly, the CFA analysis demonstrated that, while the assessment tool was originally designed for use in general population, its psychometrics confirm its applicability among mental health professionals.

These findings should be interpreted with the following limitations in mind. Firstly, the study participants were recruited through a convenience sample drawn from one single mental health institution in Singapore. Although it is the only tertiary mental health provider, the sample might not be representative of all mental health professionals in Singapore and was restricted to doctors, nurses and allied health staff and therefore this would affect the generalizability of the study findings. Secondly, since the email invitations were sent out through hospital group emails lists, it was not possible to track the exact number of staff who accessed that email; thus we are unable to provide the response rate. Thirdly, although we compared the attitudes to mental illness between mental health professionals and the general population, the two studies were conducted at different time periods through different data collection strategies which might affect their comparativeness. However, the socio-demographic characteristics were controlled for in the analysis which excluded some of the potential confounding effects. Lastly, although we examined the correlates of AMI-SG factors among mental health professionals, the cross-sectional design did not allow any casual relationships to be established. More studies are needed to further explore this topic.

## Conclusion

The current study confirmed that the AMI-SG is an effective tool to measure attitudes to mental illness among mental health professionals in Singapore, and it had the same factor structure as that among the general population. Although mental health professionals had significantly more positive attitudes to mental illness compared to the general population, their attitudes on ‘social distancing’ did not differ from the attitudes of the general population. Multivariate regression analysis suggested that attitudes to mental illness among the mental health professionals were negatively associated with Chinese ethnicity, being ever married, less educated, being a nurse, and positively associated with the status of having family or close friends diagnosed with mental illness. For future studies, researchers could 1) use more representative samples (i.e. samples across institutions providing psychiatric care); 2) explore the mechanism of how social contact affect attitudes to mental illness differently between the mental health professionals and the general public in the local context; 3) investigate the underlying mechanism of how culture might affect attitudes to mental illness among the mental health professionals; 4) explore the most appropriate level of involvement for mental health nurses in the inpatient care of individuals with mental illness in the long-stay wards.

## Supporting information

S1 Appendix Attitude to mental illness questionnaire–Singapore version (AMI-SG). (DOCX)
