# Supplementary material for: Attitudes to mental illness among mental health professionals in Singapore and comparisons with the general population
Source: PLoS One. 2017 Nov 16;12(11):e0187593. doi: 10.1371/journal.pone.0187593 (PMC5690645; doi:10.1371/journal.pone.0187593)
Supplement: S1 Appendix — (DOCX) [file pone.0187593.s001.docx]

S1 Appendix. Attitude to Mental Illness questionnaire – Singapore Version (AMI-SG)

## Factor 1 - Social distancing

AMI-SG 1 Having mental health facilities in a residential area downgrades the neighbourhood.

AMI-SG 2 It is frightening to think of people with mental problems living in our neighbourhoods.

AMI-SG 3 I would not want to live next door to someone who has been mentally ill.

## Factor 2 - Tolerance/Support for community care

AMI-SG 4 We have a responsibility to provide the best possible care for people with mental illness.

AMI-SG 5 Anyone can become mentally ill. ®

AMI-SG 6 Increased spending on mental health services is a waste of money.

AMI-SG 7 We need to adopt a more tolerant attitude toward people with mental illness in our society.

AMI-SG 8 As far as possible, mental health services should be provided through community based facilities such as policlinics, GPs and family counselling services.

AMI-SG 9 'People with mental illness are not as dangerous as most people think they are'.

AMI-SG 10 The best therapy for many people with mental illness is to be part of a community.

AMI-SG 11 Residents should not be afraid of visiting mental health services in their neighbourhood.

AMI-SG 12 No-one has the right to exclude people with mental illness from their neighbourhood.

## Factor 3 – Social Restrictiveness

AMI-SG 13 Anyone with a history of mental problems should be excluded from the public/civil service.

AMI-SG 14 People with mental illness should not be given any responsibility.

AMI-SG 15 People with mental illness are a burden on society.

## Factor 4 - Prejudice and Misconception

AMI-SG 16 As soon as a person shows signs of mental disturbance, they should be hospitalized.

AMI-SG 17 Mental hospitals are the only means of treating people with mental illnesses.

AMI-SG 18 There are sufficient existing services for people with mental illness.

AMI-SG 19 One of the main causes of mental illness is a lack of self-discipline and will-power.

AMI-SG 20 There is something about people with mental illness that makes it easy to identify them from normal people.
